# Microplastic contamination of drinking water: A systematic review

**DOI:** 10.1371/journal.pone.0236838

**Published:** 2020-07-31

**Authors:** Evangelos Danopoulos, Maureen Twiddy, Jeanette M. Rotchell

**Affiliations:** 1 Hull York Medical School, University of Hull, Hull, United Kingdom; 2 Department of Biological and Marine Sciences, University of Hull, Hull, United Kingdom; VIT University, INDIA

## Abstract

**Background:**

Microplastics (MPs) are omnipresent in the environment, including the human food chain; a likely important contributor to human exposure is drinking water.

**Objective:**

To undertake a systematic review of MP contamination of drinking water and estimate quantitative exposures.

**Methods:**

The protocol for the systematic review employed has been published in PROSPERO (PROSPERO 2019, Registration number: CRD42019145290). MEDLINE, EMBASE and Web of Science were searched from launch to the 3rd of June 2020, selecting studies that used procedural blank samples and a validated method for particle composition analysis. Studies were reviewed within a narrative analysis. A bespoke risk of bias (RoB) assessment tool was used.

**Results:**

12 studies were included in the review: six of tap water (TW) and six of bottled water (BW). Meta-analysis was not appropriate due to high statistical heterogeneity (I^2^>95%). Seven studies were rated low RoB and all confirmed MP contamination of drinking water. The most common polymers identified in samples were polyethylene terephthalate (PET) and polypropylene (PP), Methodological variability was observed throughout the experimental protocols. For example, the minimum size of particles extracted and analysed, which varied from 1 to 100 μm, was seen to be critical in the data reported. The maximum reported MP contamination was 628 MPs/L for TW and 4889 MPs/L for BW, detected in European samples. Based on typical consumption data, this may be extrapolated to a maximum yearly human adult uptake of 458,000 MPs for TW and 3,569,000 MPs for BW.

**Conclusions:**

This is the first systematic review that appraises the quality of existing evidence on MP contamination of drinking water and estimates human exposures. The precautionary principle should be adopted to address concerns on possible human health effects from consumption of MPs. Future research should aim to standardise experimental protocols to aid comparison and elevate quality.

## Introduction

Microplastics (MPs) are particles of predominantly synthetic polymeric composition in the micro scale [1, 2], and while a consensus on size range has not been reached, the typical range is between 1 μm and 5 mm. MPs have been identified in all aquatic environments: marine [3–7] and freshwater (lakes, rivers, reservoirs, groundwater) [8–15], but research has so far concentrated more on marine environments. MP contamination of aquatic environments is expected to rise, hand-in-hand with the continuous rise in plastic production, use and waste [16–20]. MPs have also entered the food web, thus becoming an emerging food safety issue and risk [21–25]. Emerging risk in terms of food safety is defined as a risk posed by possible significant exposures to a recently identified (emerging) hazard [26, 27].

Human exposure pathways include ingestion and inhalation and the presence of MPs in human stool samples has recently been verified [28]. Drinking water is considered as one possible medium for the introduction of MPs into the human body [24]. There is a growing interest around the prevalence of MPs in drinking water underpinned by recent research but a systematic review of available evidence is lacking [29–35]. None of the existing reviews have used the methodology [36] on which systematic reviews are based. Systematic reviews synthesize the findings quantitatively and qualitatively in a standardised way, avoiding the introduction of bias. Although human health effects are still under examination, lessons from toxicology inform us that the effects will be dose dependent [37–39]. Determining exposure levels is key in formulating a risk assessment framework for this emerging environmental contaminant. Health effects will be caused by: their physical attributes, the chemical properties of the polymers, the plasticisers, or other chemicals added in the manufacturing process, and the chemicals they can absorb in nature as well as the microbes that can grow on their surface [40–42].

This review focuses on water intended for human consumption, including tap water (TW) that is available to consumers via water treatment plants (WTP) and bottled water (BW). BW is further divided into table, spring and natural mineral water. Specific regulations govern their categorization according to their source and the processes that they are allowed to undergo before being bottled [e.g. 43–45]. Both natural mineral and spring water come from underground water sources, in principle, protected from pollution and are bottled *in situ*. In contrast, bottled table water can come from any source, including municipal mains (tap water), as long as it conforms to water safety specifications [43]. Water from different categories will vary in quality depending on the initial water quality, and the processes they are subjected to ensure food safety, transportation and packaging.

The aim of this review was to identify all available research on MP contamination of drinking waters and assess their quality to determine the state of the evidence and consequently, attempt quantification of human exposures in the prism of an emerging food safety issue. We also aim to compare water of different origins (TW and BW) in terms of MP contamination load. Further, we address the methodological issues in the field of environmental MPs research regarding study design, execution and reporting.

## Methods

This review follows a protocol published in PROSPERO (PROSPERO 2019, Registration number: CRD42019145290) available from: https://www.crd.york.ac.uk/prospero/display_record.php?ID=CRD42019145290 and in the S1 Protocol (available in the Supporting Information). The protocol was developed according to the guidelines set by the Preferred Reporting Items for Systematic Reviews and Meta-Analyses protocols (PRISMA-P) [46, 47]. The protocol was designed to include available research on all food categories which were determined by a preceding scoping review. In brief, only descriptive and analytic observational study designs (and not experimental) were included [48]. No time limit on publication date was set and databases were searched from launch date to 10^th^ July 2019. The searches were repeated on the 3rd of June 2020 to include the most recently published papers. Only studies that reported on ‘water intended for human consumption’ as defined by Directive 2009/54/EC [44] and Regulation (EC) No 178 [49] were included. Eligible studies must have used one (or more) of the four currently validated processes for the identification of microparticle composition: Fourier-transform infrared spectroscopy (FTIR), Raman spectroscopy (RM), pyrolysis gas chromatography/ mass spectrometry (Pyr-GC-MS) and scanning electron microscopy plus energy-dispersive X-ray spectroscopy (SEM/EDS). The use of procedural blank samples was also mandatory. Articles that were not published in the English language were excluded.

Information sources were MEDLINE (OVID interface, 1946 onwards), EMBASE (OVID interface, 1974 onwards) and the Web of Science core collection (Web of Science, 1900 onwards). The search strategy was developed for MEDLINE and EMBASE (OVID interface) using free text and MeSH, for all food categories. Search terms included: microplastic, nanoplastic, food contamination, water contamination (full search strategy can be found in S1 Table). Study selection was executed using a two-level screening by two independent reviewers against the inclusion/exclusion criteria. Any discrepancies were resolved by a third-party arbitrator. Inter-rater agreement level for the first level screening was 90%, Cohen’s k: 0.34, and for the second level: 100%, Cohen’s k: 1 [50]. A form previously developed and verified for a scoping review was used for data extraction.

The quality of the studies was assessed with the use of a bespoke risk of bias (RoB) assessment tool, which was developed because the existing tools were not suitable for the scope of the review [51]. Assessment tool development was based on guidelines set by the Centre for Reviews and Dissemination [52], the STROBE Statement checklist [53], the Agency for Healthcare Research and Quality of the U.S. Department of Health and Human Services [54], the Environmental-Risk of Bias Tool [55] regarding evidence in environmental science and the Cochrane Collaboration’s tool for assessing RoB [56]. The RoB tool, is a checklist (S2 Table), that prompts questions across four domains: study design, sampling, analysis and reporting, leading to an overall assessment with justification for each entry [57]. There were three ratings: high risk, low risk or unclear RoB and the results were used to assess study quality and overall certainty of evidence.

The primary outcome of interest was MP content in the sample expressed in a quantitative measure in any available units of measurements. Further information of interest included the methodological specifications of the experimental protocols. The studies were reviewed in a narrative analysis according to the guidelines set down by the Centre for Reviews and Dissemination [52] and the results were reported according to the Preferred Reporting Items for Systematic Reviews and Meta-Analyses (PRISMA) Statement [58, 59] (S1 PRISMA Checklist).

## Results

### Study selection

**Fig 1:**
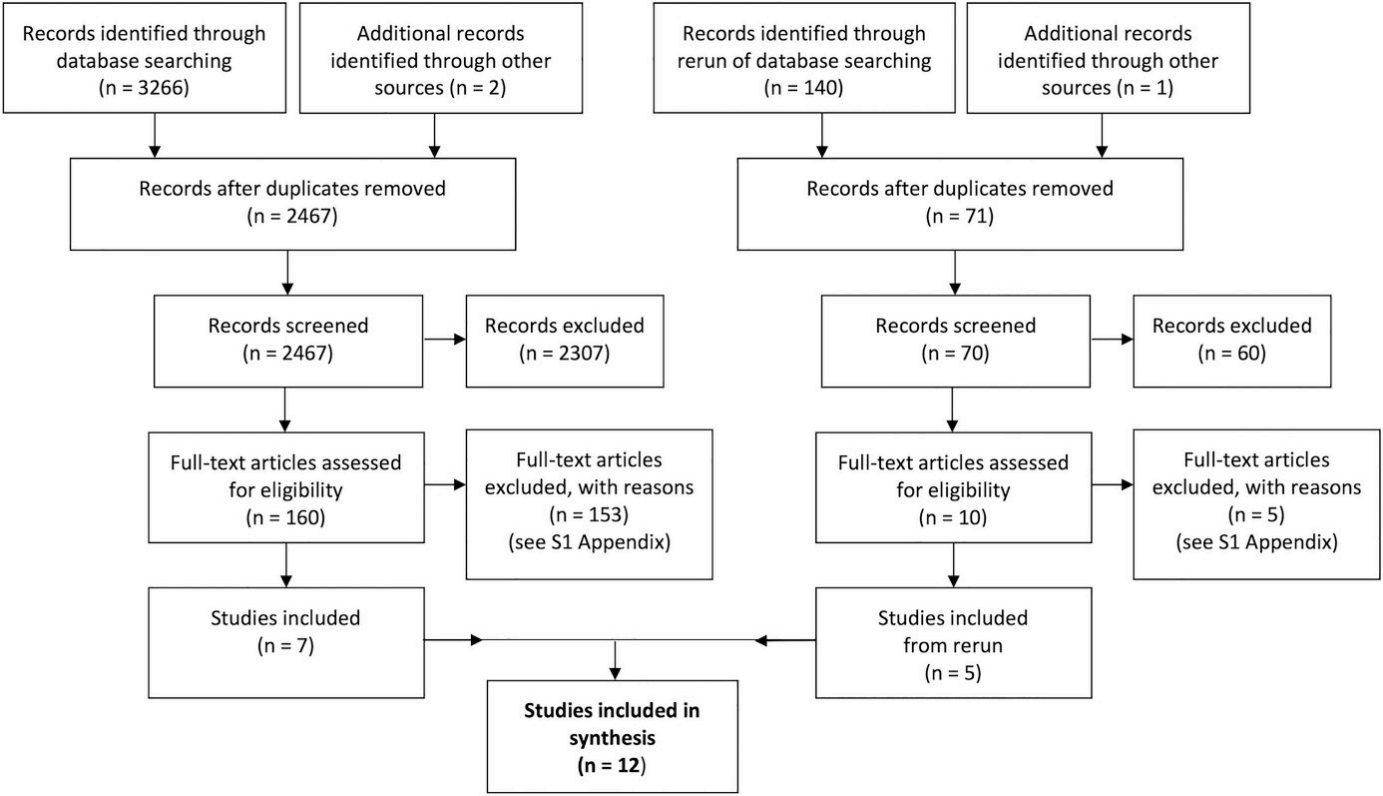
PRISMA flow chart. The flow chart presents the results and screening process of the original searches and the rerun of the searches.

2467 citations were identified by the search strategy, after duplicates were removed, and 2307 citations were dismissed in the first-level screening based on their title and abstract (Fig 1). During the second-level screening, the full papers were scrutinized, and 112 studies were removed with reasons (S1 Appendix) and seven were included. When the searches were re-run, five more studies were included after the first and second level screening (Fig 1), resulting in 12 studies [60–71] finally included in this systematic review.

### Study characteristics

All the studies included analysed water readily available for human consumption. The study characteristics are presented in S3 Table. Six studies used samples of BW (table and mineral) and six studies used TW. The overall sample size for BW was n = 91 brands (n = 435 bottles) and for the TW, n = 155 samples. All of the studies used different techniques to extract particles from their samples. One study used FTIR [61], three studies used m-FTIR [62, 67, 70], one study used RM [68], four used m-RM [63, 65, 66, 69], one both FTIR and RM [60], one used both m-FTIR and m-RM [64] and one SEM-EDX [71] to identify the composition of the extracted particles. Ten of the studies reported the results by MP particles per volume, one provides only the range of MP content and one the frequency of occurrence.

### Risk of bias within studies

**Fig 2:**
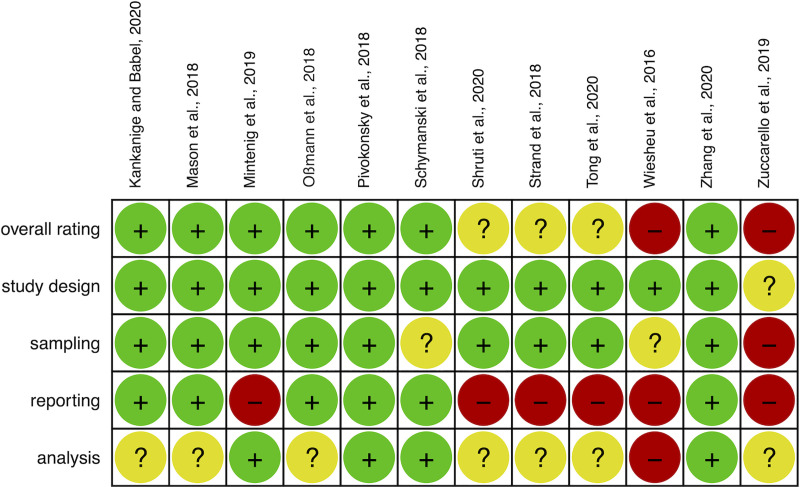
RoB assessment in individual water studies. The figure shows the rating for the four domains and the overall rating for each study. Red (-) indicates high RoB, green (+) indicates low RoB and yellow (?) indicates unclear RoB (Unclear RoB is given to a study when substantial information to make an informed assessment have not been reported).

**Fig 3:**
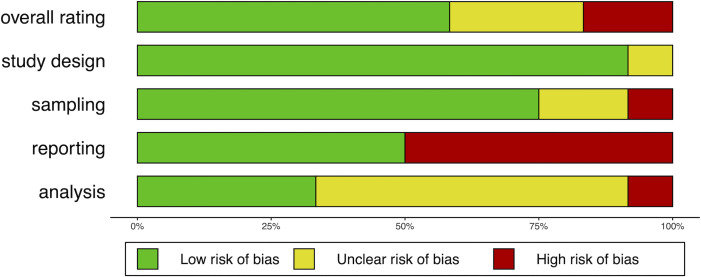
RoB assessment across all water studies.

RoB was assessed in a systematic way using the RoB tool created for this review. The results of the assessment are illustrated in Figs 2 and 3. Two studies were assessed as of high RoB [69, 71] and three of unclear RoB [66–68]. The RoB assessment is used in the analysis part of the review.

### Results of MPs contamination

The results are presented in Table 1 as two categories of TW and BW. The results from Mintenig et al. [62] were converted from MPs/m^3^ to MPs/L content for ease of comparison to the remaining studies. Mason et al. [61] divided the results in two sections: one including particles ≥100 μm that were verified as MPs through FTIR spectral analysis and particles <100 μm that were only tagged using Nile Red solution to dye them. In line with our eligibility criteria, only the results of the FTIR verified particles will be included in this review. Visual observation for the identification of MP particles can lead to under or overestimations [31]. The use of instruments which identify the chemical composition in a standardized way based on a physical or electronic output (spectra, pyrograms etc.) exclude the introduction of human error and enable reproducibility and transparency of the results.

**Table 1:** Drinking water studies results.

Study, Year	Sample type	N		Sample volume	MPs/L	±SD	Range MPs/L	% of Samples containing MPs	Polymers	Shape
Mintenig et al. [62] (2019)	TW: Ground-water from wells	N = 24	n = 9 raw, n = 15 drinking	8,000, 32,000 L	0.0007		0–0.0007	42%	Polyester 62%, PVC 14%, PA and epoxy resin 9%, PE 6%	fragments^a^
Pivokonsky et al. [64] (2018)	TW: from WTPs^b^	N = 36	WTP1 n = 12	1 L per sample	443	10		100%	PET 41%, PP	fragments > fibres > sphericals
			WTP2 n = 12		338	76			PET 62%, PP	
			WTP3 n = 12		628	28			PET 26%, PP, PE 24%	
Shruti et al. [66] (2020)	TW	N = 42	metro stations water fountains	3 L x 3 per site	18	7	5 ± 2 to 91 ± 14	100%	PTT, epoxy resin	fibres > fragments
Strand et al. [67] (2018)	TW	N = 17	n = 9 private households	50 L for each sample	< 0.58			24%	PP 50%, PS 25%, PET 25%	fragments
			n = 3 private workplaces							
			n = 5 private or public institutions							
Tong et al. [68]	TW	N = 38	private households	2 L per site	440	275	0 to 1247	95%	PE 26.8%, PP 24.4%, co PE-PP 22.0%, PPS 7.3%, PS 6.5%, PET 3.3%	fragments > fibres > spheres
Zhang et al. [70] (2020)	TW	N = 7	private households	4.5 L x 3 per site	0.7	0.6	0.3 to 1.6	100%	Rayon, PET, PE, PS, Polyester, PAA, PMPS, PI	fibres > fragments
Kankanige and Babel [60] (2020)	BW: Spring and tap	10 brands, 95 bottles	n = 65 PET single use bottles	10 brands: total 43.23 L	140	19		100%	PET 28.4%, PE 24.2%, PP 18.1%, PA 7.2%, PVC 4.4%	fibres > fragments
			n = 30 glass bottles		52	4				
Mason et al. [61] (2018)	BW: table and mineral	11 brands, 259 bottles	n = 253 plastic bottles	9 brands: 500–600 ml per bottle, 2 brands: 0.75–2 L per bottle	10.4^c^ (≥100 μm), 315 (6.5–100 μm)		0–14	93%	PP 54%, Nylon 16%	fragments > fibres > films
			n = 6 glass bottles							
Oßmann et al. [63] (2018)	BW: mineral	21 brands, 32 bottles	n = 12 PET reusable bottles	0.5–1 L per bottle	4889	5432		Not specified	PET, PP, PET and olefin, PE	Not specified
			n = 10 PET single use bottles		2649	2857			PET, PET and olefin, PP, PE	
			n = 9 glass reusable bottles, n = 1 glass single use bottle		6292, 3074^d^	10521, 2531^d^			PE, PP, Styrene-Butadiene, PET	
Schymanski et al. [65] (2018)	BW: mineral	38 brands, 38 bottles	n = 15 returnable plastic bottles	700–1500 ml	118	88	28–241	100%	PET 84%, PP 7%, PE 5%, PA 2%	fragments
			n = 11 single-use plastic bottles		14	14	2–44			
			n = 3 beverage cartons		11	8	5–20			
			n = 9 glass bottles		50	52	4–156			
Wiesheu et al. [69] (2016)	BW: mineral	1 brand	n = 1	3 L	1 in the sample^e^			Cannot confirm contamination	PET	fibres
Zuccarello et al. [71] (2019)	BW: Mineral still and sparkling	10 brands, 10 bottles	N = 10 plastic bottles	500 ml per bottle	5.42 X 10^7^	1.95 X 10^7^	3.16 X 10^7^ to 1.1 X 10^8^	100%	Not specified	Not specified

^a^ fibres were not taken into consideration.

^b^ Water Treatment Plant.

^c^ only particles ≥100 μm were verified with FTIR.

^d^ without outlier.

^e^ only fibres counted.

PP polypropylene, PVC polyvinyl chloride, PA polyamide (nylon), PE polyethylene, PET polyethylene terephthalate, PS polystyrene, PTT poly trimethylene terephthalate, PPS polyphenylene sulphite, PAA polyacrylic acid, PMPS poly (methyl phenyl siloxane), PI poly (isoprene).

Regarding studies other than BW, when results were presented for both untreated and treated water, only the latter are presented.

### Tap water

Six studies [62, 64, 66–68, 70] sampled and analysed TW that was readily available to consumers via a public service. The percentage of samples containing MPs across the studies ranged from 24% to 100% and the MPs content from 0–1247 MPs/L. The most common shapes identified were fragments and second most common was fibres. A key difference between the samples is that Pivokonsky et al. [64] used water coming from surface waters (reservoirs), which are open aquatic systems exposed to contamination, while Mintenig et al. [62] used water from underground and therefore protected sources. Shruti et al. [66] used water from a variety of sources but the majority came from local aquifers. Strand et al. [67], Tong et al. [68] and Zhang et al. [70] did not provide information on the origin of the water. It is reasonable to assume that water quality before it entered the WTP would vary and directly affect the quality of the water after processing [8].

Four of the studies [64, 66, 68, 70] provided the necessary data to attempt a meta-analysis. In order to test whether the results were appropriate for meta-analysis, the statistical heterogeneity was measured using a Higgins I^2^ test [72], calculated using R (version 3.6.0) [73], executing all analysis via RStudio, (version 1.2.1335) [74], and using the additional packages meta (version 4.9–7) [75], metaphor (version 2.1–0) [76], dmetar [77], robvis [78] and ggplot2 [79]. A random-effects model was fitted [80, 81] and heterogeneity was found to be high, I^2^ = 99.8% (see forest plot in S1 Fig). In order to detect the origin of heterogeneity, a series of random-effects models were fitted excluding two studies [64, 68] that were identified as statistical outliers. The exclusion of the studies did not improve heterogeneity which remained high (100%). Therefore, the data were found to be inappropriate for meta-analysis. Heterogeneity is either caused by clinical (sample) or methodological variability [36, 82] and is further discussed in the narrative analysis section.

#### Sample treatment/particle extraction

The experimental protocol for the extraction of particles differed between the six studies in terms of sample collection, treatment and filtering. Mintenig et al. [62] filtered the water directly at the sampling sites using stainless steel filter cartridges (3 μm) and then further treated the residue on the filters at the lab. A solution of hydrochloric acid was used to dissolve inorganic material, such as calcium carbonate and iron precipitates, followed by a second filtering through another 3 μm stainless steel filter. The residue was treated again using hydrogen peroxide before the third and final filtration on 0.2 μm aluminium oxide filters. An additional density separation step was used for the raw water samples, employing a zinc chloride solution to remove further iron oxide particles. Strand et al. [67] also filtered the samples at the sampling sites but using a stainless-steel filter with absolute filtering ability of 11–12 μm. The sample was then treated using a solution of acetic acid. For the collection of the particles used for the spectral analysis, a backwashing procedure with detergent solution was used, this was pre-filtered water and then ethanol under vacuum suction on an Anodisc filter (0.2 μm). Four studies [64, 66, 68, 70] collected the samples in bottles and then transported them to the lab for processing. Pivokonsky et al. [64] used wet peroxide oxidation and heat treatment at 75°C for digestion, followed by a double filtration through 5 μm and then 0.2 μm membrane filters (PTFE). Tong et al. [68] used hydrochloric acid for digestion followed by filtering through 0.2 μm aluminium oxide filters. In contrast, Shruti et al. [66] and Zhang et al. [70] did not treat the samples prior to filtering, using 0.22 μm and 0.45 μm pore size filters respectively.

The difference in the pore size of the filters used in the different stages reflects the sizes of the particles extracted which were subsequently further analysed for composition identification, and has thus directly affected the measured MP content. On the other hand, the use of a digestion step to dissolve particulate matter is employed only by some of the studies to extract water impurities and optimize the filtration process.

#### Spectral analysis

Differences in the methodology of the studies were identified while important information such as the number of extracted particles and the number of particles that were analysed for composition were not reported (Table 2). Three studies used FTIR for spectral analysis, while Pivokonsky et al. [64] also used RM for the smaller size range of 1–10 μm. One study used m-FTIR, one RM and one m-RM. A key difference between them is the technical limitation of the instrument regarding the minimum particle size detected. FTIR and RM technical specifications are in the range of 40 μm and 10 μm, respectively. When these methods are used in conjunction with microscopes, it becomes possible to analyse particles down to the size of 10 μm (m-FTIR) and 1 μm (m-RM) [31, 83–86]. Mintenig et al. [62] and Zhang et al. [70] analysed 100% of the filters’ surface, Pivokonsky et al. [64] about 25% of the sample and Strand et al. [67] 10% of the filter but coming from only three out of the 17 sampling sites/samples. Shruti et al. [66] and Tong et al. [68] did not report the amount of the sample analysed.

**Table 2:** Particle identification specifications for tap water studies.

Study	Filter pore size	Method	Min size for spectral analysis	Particles extracted	Particles for analysis	% for analysis	Spectral similarity index	Verified MPs
Mintenig et al. [62]	3 μm, 0.2 μm	FTIR	≥20 μm	n/s^a^	n/s	100%	n/s	n/s
Pivokonsky et al. [64]	5 μm, 0.2 μm	RM	1 μm	n/s	n/s	~25%	80%	n/s
		FTIR	≥10 μm					
Shruti et al. [66]	0.22 μm	m-RM	500 μm	n/s	n/s	n/s	n/s	n/s
Strand et al. [67]	~12 μm^b^, 0.2 μm^c^	FTIR	≥10 μm	n/s	n/s	10% of 3 out of 17 samples.	n/s	3%
Tong et al. [68]	0.2 μm	RM	n/s	n/s	n/s	n/s	n/s	n/s
Zhang et al. [70]	0.45 μm	m-FTIR	>10 μm	n/s	n/s	100%	70%	n/s

^a^ not specified.

^b^ for MP content.

^c^ for spectral analysis.

None of the studies reported the final number of particles analysed and only Strand et al. [67] reported the success rate of conclusive identification (44%) and the proportion that was identified as MPs (3%). Only the two studies by Pivokonsky et al. [64] and Zhang et al. [70] reported the similarity index for the spectral analysis, 80% and 70%, respectively. Although scientific guidance on the particles that need to be analysed does not exist, it is reasonable to assume that larger proportions would lead to more robust results. Mintenig et al. [62] did not analyse the fibres at all. Although a larger number of fibres were discovered compared with ‘particles’ in the samples, spectral analysis was not utilised because the fibre presence was attributed to their presence as post-sampling contamination. Fibres are a high proportion of MPs and their complete exclusion from the results might have resulted in an underestimation of MP content.

#### Particle size

**Fig 4:**
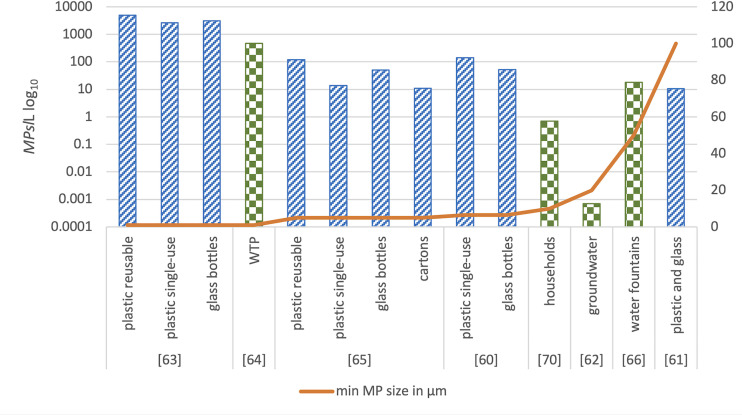
MP content in TW and BW. MP content (MPs/L) is illustrated in the left-hand side y axis in log_10_ scale. BW: diagonal stripes, TW: chequerboard, Minimum particle size included in each study is illustrated in the right-hand side y axis. Studies by Tong et al. [68], Wiesheu et al. [69] and Zuccarello et al. [71] were not included because they were rated as of high RoB.

The key difference in the studies’ protocol is the size of the particles identified and verified via spectral analysis and is directly connected to the extraction process and the composition identification process used. Shruti et al. [66] only analysed particles >500 μm, Mintenig et al. [62] ≥20 μm, Strand et al. [67] and Zhang et al. [70] ≥10 μm, Pivokonsky et al. [64] ≥1 μm, while Tong et al. [68] did not report the minimum size. The study by Pivokonsky et al. [64] reported the highest MP content ranging from 338 ±76 to 628 ±28 MPs/L and stated that 25–60% of the MPs were in the range of 1–5 μm and 30–50% in the range of 5–10 μm. Tong et al. [68] reported content in the same magnitude of 440 ±275 MPs/L, and state that MPs <50 μm were significantly dominant. It must be noted that Tong et al. [68] used only Nile Red dying and visual identification for the determination of particle size in a reported range of 3–4453 μm. The results from these two studies present a noteworthy difference. When the MPs’ size range is taken into consideration it becomes clear that this variance could be attributed to the fact that the other four studies were not able to detect that same range of sizes (Fig 4). In addition, it should be noted that although Strand et al. [67] state that particles were measured down to 10 μm, the majority of the results were based on particles ≥100 μm. The inverse relationship between the size of MPs and their abundance is further supported by the findings of Shruti et al. [66] who reported that 75% of the particles were in the range of 100 μm– 1 mm, Zhang et al. [70] who reported that 46% were in the range of 500 μm -1 mm and Mintenig et al. [62] who found that all particles were in the range of 50–150 μm.

### Bottled water

Six studies samples BW (Table 3). Kankanige and Babel [60] sampled spring and TW, Mason et al. [61] sampled table and mineral water and the rest of the studies sampled only mineral water. Three different container materials were selected: plastic (single-use and reusable), glass and carton. MPs content ranged from 0 to 1.1 X 10^8^ MPs/L across all containers. The percentage of samples containing MPs ranged from 92% to 100%. Fragments and films were the most commonly identified shape.

**Table 3:** Particle identification specifications for bottled water studies.

Study	Filter pore size	Method	Min size for spectral analysis	Particles extracted	Particles for analysis	% for analysis	Spectral similarity index	Verified MPs
Kankanige and Babel [60]	0.45 μm	FTIR	≥50 μm	839	839	100%	60%	45.8%
		RM	1–50 μm	n/s^a^	n/s	n/s	n/s	n/s
Mason et al. [61]	1.5 μm	FTIR	≥100 μm	n/s	~1000	~50%	70%	40%
Oßmann et al. [63]	0.4 μm	RM	≥1 μm	n/s	n/s	4.4% of each filter area	n/s	n/s
Schymanski et al. [65]	3 μm	RM	≥5 μm	n/s	~1000^b^	100%	70%	0.03 to 10.7%
Wiesheu et al. [69]	0.45 μm	RM	≥1 μm	n/s	1	100%	n/s	n/s
Zuccarello et al. [71]	n/a^c^	SEM-EDX	0.5 μm	n/a	n/a	0.2% of each stub area	n/a	n/a

^a^ not specified.

^b^ for each sample in the 5–10 μm size fraction.

^c^ not applicable.

Meta-analysis was attempted using the results from four of the studies [51, 60, 65, 71] which provided the necessary data. Statistical heterogeneity as measured by Higgins I^2^ test [72] in a random-effects model was found to be high, I^2^ = 99%, even when the high RoB study by Zuccarello et al. [71] was excluded (S2 and S3 Figs). Examining the four different types of containers separately in a mixed-effects subgroup analysis [80, 81], statistical heterogeneity within the groups still remained high I^2^<84% (S4 Fig). The pooled effect estimate was accompanied by a 95% confidence interval which included negative values for all categories, further showing that meta-analysis was not appropriate. The results of the analysis showed that pooling of the data was not appropriate. The origin of heterogeneity is addressed in the narrative analysis.

#### Sample treatment/particle extraction

Four studies [60, 61, 65, 69] did not use a digestion process. Mason et al. [61] used glass-fibre filters (1.5 μm pore size), Schymanski et al. [65] used gold-coated poly-carbonate filters (3.0 μm pore size) while both studies by Kankanige and Babel [60] and Wiesheu et al. [69] used cellulose nitrate filters (0.45 μm pore size). Oßmann et al. [63] implemented a digestion process using an ethylene diamine tetra-acetic acid tetrasodium salt (EDTA) solution then followed by a density separation (flotation) step via a detergent solution of sodium dodecyl sulphate (SDS) and filtration through aluminium-coated polycarbonate membrane filters (0.4 μm pore size). Zuccarello et al. [71] did not employ a digestion nor a filtration process, opting for a newly developed method to target MPs <10 μm, which differs significantly from previous studies and cannot thus be directly compared to the rest of the studies. The alternative approach used nitric acid and a high temperature incubation (60° C for 24 hours) for mineralization of the samples to remove carbon-based particles. This was followed by vortexing, centrifugation, addition of dichloromethane, resuspension using acetonitrile and drying. The sample was then deposited on an aluminium and copper alloy stub to be coated with gold before SEM-EDX analysis [87]. The methods used by this study have already been highlighted [88] under the reporting and verification sections of the analytical methods which was partially addressed by a corrigendum of the authors [87]. The scientific base of the process employed is a publication that is not available in English [89] and therefore cannot be assessed, as well as a second publication [90] concerning MPs extraction method from the gastrointestinal tract of fish. The latter describes a different method (two-step digestion process using sodium hydroxide and nitric acid, followed by filtration, density separation and verification by visual identification alone, that subsequently targets MPs of a completely different size of >100 μm).

#### Spectral analysis

Schymanski et al. [65] examined the largest number of particles in RM spectral analysis, analysing 100% of the particles or a maximum of 1000 (in the 5–10 μm size fraction) on each of the filters, corresponding to each of the 38 samples (Table 3). The verified MP particles ranged from 0.03 to 10.7% of the analysed particles, using a ≥ 70% spectral similarity index. Kankanige and Babel [60] analysed 100% of the extracted particles (>50 μm), using FTIR and a 60% spectral similarity index, verifying 45.8% of them as MPs. RM analysis was used for particles of the lower range of 1–10 μm but these findings are not reported in the details of the analysis. Mason et al. [61] also used FTIR but only for particles ≥100 μm and examined around 1000 particles which was almost 50% of the particles extracted, using a ≥ 70% similarity index verifying 40% of the particles as MPs. Oßmann et al. [63] on the other hand, did not provide information on the number of extracted particles, reporting the analysis of 4.4% of the surface of each filter using RM, but not reporting how many were finally verified as MPs. Oßmann et al. [63] did not use an automated software option in which spectral similarity is calculated automatically but a mix of semi-automated methods. In this sense, a standardized spectral similarity index was not utilised, which might have introduced experimental error into this protocol. Wiesheu et al. [69] only analysed the one fibre extracted from the samples isolated, not providing further details on the methods employed.

Zuccarello et al. [71, 87] used SEM-EDX for the identification of MPs. No digestion or filtration process for the extraction of the mineral water impurities was employed. The authors suggest that the mineralization process extracts all carbon-containing particles that are not plastic. This removal needs to be done with near unit efficiency due to the fact that typical concentrations of carbonates in mineral water exceed, by many orders of magnitude, the reported MP concentrations in BW samples in other studies. The specificity of this method has not been proven as mentioned in the previous section. The aim of the method was to quantify the number of MPs per volume in the size range of 0.5–10 μm and a further objective was to calculate the mass of MPs per volume, using the density of the plastic bottles containing the water. The reported validation of the process used is weak in that the mass of MPs per volume was measured in three samples spiked with MPs (whose size was not reported), and then a calculation of MPs per volume was conducted, which is the opposite way round to the calculation made with the unknown samples and may introduce systematic error.

#### Particle size

Mason et al. [61] used FTIR only for particles ≥100 μm but reported that 95% of particles were between 6.5 and 100 μm. The MP content for all sizes was 325 MPs/L, whereas for particles ≥100 μm it was only 10.4 MPs/L. In addition, it was not clear what maximum size cut-off was employed. Kankanige and Babel [60] used FTIR for particles ≥50 μm but extrapolated the findings to the smaller size range 6.5–50 μm, reporting MPs contents of 140 ±19 MPs/L for plastic bottles and 52 ±4 MPs/L for glass bottles. The size range of 6.5–20 μm was identified as the most dominant. Schymanski et al. [65] extracted and analysed particles including even smaller sizes of ≥5 μm and reported that 80% of the verified MPs were in the range of 5 and 20 μm, with MP contents of 14 ±14 MPs/L for single use plastic bottles, 118 ± 88 MPs/L for reusable plastic bottles, 11 ± 8 MPs/L for carton and 50 ± 52 MPs/L for glass bottles. Oßmann et al. [63] decreased the size of the included particles to ≥1 μm reporting much higher MP contents of 2649 ± 2857 MPs/L for single use PET bottles, 4889 ± 5432 MPs/L for reusable PET bottles and 6292 ± 10521 MPs/L for glass bottles. The same authors also highlight that more than 95% of MPs were smaller than 5 μm and 50% smaller than 1.5 μm. Zuccarello et al. [71] focused on the 0.5–10 μm size range, reporting high concentrations of 5.42 ± 1.95 X 10^7^ MPs/L. Although the size range of the identified MPs (1.28–4.2 μm) is similar to the Oßmann et al. [63] study (>1 μm), the results differ by a factor of 11000, further highlighting the possible quality issues of the study. The results of the Wiesheu et al. [69] study on MPs content were inconclusive. As can be seen in Fig 4, as the size of the identified particles decreases, the MP content increases significantly.

## Discussion

Twelve studies were systematically reviewed, which collectively analysed more than 40000 L of TW and 435 bottles of BW (table and mineral water). It would not be reasonable to collate the evidence from the twelve studies included in this systematic review due to key differences that were identified in the experimental protocols and high sample heterogeneity. In addition, the lack of key information (e.g. SE, SD) and high statistical heterogeneity hinder the execution of meta-analysis in an attempt to quantify MP content. RoB was found to be low in the majority the studies. Two studies were rated as of high RoB and therefore the results of these are excluded. The study by Zuccarello et al. [71] was rated high RoB in the two domains of sampling and reporting, while the study by Wiesheu et al. [69] was rated high RoB in the domains of analysis and reporting.

All studies reported some level of MP contamination. Samples positive for contamination ranged from 24–100% in TW and 92–100% for BW. Comparing the results between the different water origins, specifically between the two studies [63, 64] that targeted similar MP sizes of minimum 1 μm, MP content was higher in BW (plastic and glass bottles) than TW (Fig 4). Therefore, current evidence suggests that there are higher rates of MP contamination in BW compared with TW, both in terms of frequency and quantity. Regarding the primary origin of BW, Mason et al. [61] analysed table and mineral BW and Kankanige and Babel [60] tap and spring BW, but did not report a comparison between the different water origins which could shed some light on the possible differences.

The methodology used in the studies varied in both sampling and analysis. Standardization of the experimental protocols is key in order to increase confidence in the quality of the studies and certainty of the evidence. The first step in obtaining comparable and trustworthy results is the use of a verified composition identification process, which was employed by all of the studies included in this review. Not using such a process has been proven to lead to gross under- or over-estimations [31, 91, 92]. Even with all the studies using either FTIR, RM or SEM-EDX, there were still differences in the spectral similarity index, the number and proportion of the particles analysed, and the spectral library used. Furthermore, poor reporting hindered the assessment of the experimental protocols’ effectiveness; only one study [60] reported how many particles were retrieved from the extraction process and only four [60, 61, 65, 69] reported how many particles were analysed for composition identification.

The most significant difference in the methods is the size of the particles that were extracted from the samples and analysed for composition identification. Studies using FTIR and RM were able to analyse particles down to 1 μm which significantly influenced the results. The degradation of MPs in the marine environment and the exponential increase of the number as the size decreases has been experimentally and mathematically explored [93–95]. This would suggest that the same fragmentation pattern may also apply to other aquatic environments as well.

On the other hand, only seven [62–64, 66, 68–71] of the twelve studies reported the upper limit of the range in MP size. The importance of defining and reporting the size range of the identified MPs has a double significance as follows. As a methodology parameter it is connected to the quantified MP content results. As a food contamination parameter, it is indicative of the potential health effects. MPs <1.5 μm are characterized as more dangerous since they are, in theory, capable of crossing the gut epithelium, further progressing into the human body and thus possibly causing an adverse health effect [23].

Differences in sample size were striking, ranging from 36 to 32000 L (per study) for TW and 3 to (>)130 L for BW. At the moment, methodological consensus concerning sample size does not exist. Koelmans et al. [30], in a recent review, proposed a minimum of 1000 L for TW and 500 L for BW. In the first instance, sample size is dictated by the objectives and design of the study which in many cases are a function of the available resources [96, 97]. Sample size should be directly connected to the contaminant under examination. The volume of the samples as well as the sampling frequency can only be set when there is enough evidence to support what a meaningful MP content is. Meaningful being expressed in terms of food safety linked to human health and what is considered to be ‘wholesome and clean’ water intended for human consumption, which is the requirement of relevant European regulations and universal standards [43, 49, 98]. At the moment, there is not enough evidence to formulate an informed guideline for sampling sizes, nevertheless scientific experience points to larger sample sizes being more robust and reliable [99].

Another area of importance is quality assurance of sampling and sample handling to avoid cross contamination via airborne MPs. This issue was addressed by our RoB assessment tool in the sampling domain. In addition, only studies that employed blank procedural samples to account for this type of experimental error were included [100, 101]. The lack of detailed information on the results and the significance of procedural blank samples downgraded the quality of the study as assessed by the RoB assessment tool. The bespoke RoB tool used did not employ scales to rank the studies as done by other reviews in the field [30] but is a domain-based evaluation according to the guidance of leading methodology regarding systematic reviews [36]. The use of scales in RoB assessment is explicitly discouraged as research experience has shown that they can be unreliable [57].

Seven studies used samples from Europe (3 TW, 4 BW), three from Asia (2 TW, 1 BW), one from North America (TW), and one from multiple continents (BW) (S3 Table). The highest MPs content are reported in Europe for both TW and BW. Regarding TW, the highest reported MPs content for Europe and Asia were in the same magnitude but almost 25 times higher than those reported for the samples from North America. In BW, the maximum reported MPs content in Europe was 35 times higher than that reported in Asia. However, it is not clear if this is due to the number of existing studies and the varying methodology employed, or the geographical location. Recent research has shown that MP contamination of the environment is directly linked to waste management, which is compromised in developing countries [102, 103]. In this sense, it would be reasonable to expect higher MPs contamination of potable water in these countries, where further research is needed. In terms of polymeric composition, PET and PP were the most prevalent polymers identified in BW. The differences between the polymeric composition in the various BW studies can be attributed to the different origin of the water, processing, the material used for packaging but also to the different particle sizes the studies extracted and analysed since degradation rates between polymers vary [2, 104]. In TW, polymeric composition varied with PET and PP present along with polyester, PTT and rayon. This may possibly due to the wide geographical and environmental origin of the water samples. Rayon is a man-made but not synthetic fibre and is not included in most MP research. It should be noted that the most produced and used polymers for the last 15 years have been PE and PP, whose prevalence would be anticipated to be the highest in terms of environmental contamination although geographical variation is expected [17–20]. Fragments and fibres were the prevalent MP shape in both categories, highlighting an agreement in the findings across all studies. Polymeric composition and shape characteristics can be used as guides to the origin of MPs as well as to focus future toxicological research.

A recent review by Koelmans et al. [30] has recently addressed the issue of MPs contamination of drinking water. Koelmans et al. [30] focused not only on drinking water but also on freshwater MP contamination and experimental methodology and did not attempt quantitative collation of the evidence. The study assessed the quality of the studies using a bespoke rating system, focusing on different aspects of experimental design and execution using a scoring system. The use of scoring scales in quality assessment is explicitly discouraged by the Cochrane Collaboration, which is the leading body of systematic reviews, as research experience has shown that they can be unreliable due to the lack of justification for the ratings [36, 51, 57, 105]. The World Health Organization (WHO) delivered a report [24] based on a commissioned systematic review by Koelmans et al. [30], yet the authors make no claim that it is systematic, nor is there a description of the relevant review methods utilised, such as the existence of a published protocol.

### Human MPs exposure via drinking water

Water intake varies in adults depending on gender, climate, diet and physical activity. The WHO guideline value for water daily consumption is 2 L for adults (with a default body weight of 60 kg), 1 L for children (default body weight of 10 kg) and 0.75 L for infants (default body weight of 5 kg) [98]. Maximum daily human exposures were calculated by using the highest MP content evidence that have been rated of low and unclear RoB for the three continents, and the WHO values for daily water consumption and use [98]. The highest daily possible exposures were calculated in Europe at 1260 MPs for TW and 9800 MPs for BW (Table 4). These exposures are significant underestimations since they assume that all populations have access to treated drinking water which is not the case. These high exposure levels are driven more by the amount of water we consume and less the absolute MP content of water compared to other food categories.

**Table 4:** Maximum daily and yearly MP uptake via water direct and indirect consumption per capita.

			Adults ^a^		Children ^b^		Infants ^c^	
Continent	TW/BW	Max MPs/L	Daily MP uptake	Yearly MP uptake	Daily MP uptake	Yearly MP uptake	Daily MP uptake	Yearly MP uptake
Europe	TW	628 [64]	1256	458440	628	229220	471	171915
	BW	4889 [63]	9778	3568970	4889	1784485	3667	1338364
Asia	TW	440 [68]	880	321200	440	160600	330	120450
	BW	140 [60]	280	102200	140	51100	105	38325
North America	TW	18 [66]	36	13140	18	6570	14	4928
	BW	10.4 [61]^d^	21	7592	10	3796	8	2847

^a^ Adults: 2 L water/day, default body weight 60 kg.

^b^ Children: 1 L water/day, default body weight 10 kg.

^c^ Infants: 0.75 L water/day, default body weight 5 kg[98].

^d^ The results of the Mason et al. [61] study were used since it was the only that sampled brands of BW from multiple continents including America (n = 3).

After the ingestion of MPs, particles <1.5 μm could pass the gut barrier and translocate to other organs. Paradigms from studies on plastic material that have been used for orthopaedic replacement prosthetics have proved translocation of plastic particles to organs such as the liver, spleen and lymph nodes [106–109]. The effects of MPs will depend on their size, polymeric composition, additives (plasticisers), the chemicals that they might have absorbed from the environment, their chemical state and where they are located in the human body [41, 110].

An additional possible exposure pathway that has not yet been investigated may occur from the use of MP contaminated water for incorporation into food. According to WHO estimations, 7.5 L of water per capita per day [98] is used by most people in most situations around the world for hydration and incorporation into food. This is a complex issue since it is not clear to what extent MPs in the water would be taken up into the foodstuffs. This would depend on how the food is prepared and have geographic, cultural variation. Nevertheless, further research into this issue is clearly warranted as it is another potential pathway for MPs in water to enter the human body.

### Strengths and limitations

To our knowledge this is the first systematic review focusing on MP contamination of water intended for human consumption. The review was based on a protocol which was created beforehand, outlining the methodology used throughout. The protocol ensures that bias is not introduced. In addition, the quality of studies was assessed using a systematic RoB tool tailored to the needs of the review, addressing every stage of design, execution and reporting of research. The review was limited to a narrative analysis and did not include a meta-analysis due to high sample, experimental and statistical heterogeneity as well as poor reporting in a fraction of the studies. The majority of the studies were assessed to be low RoB.

## Conclusions

Research methodology in the field of MPs environmental contamination has advanced in recent years, especially with the use of FTIR and RM validation of particle characteristics, but is still lacking in quality and robustness. The systematic review identified specific areas where further development and standardization is needed:

- Sampling methodology: sampling size, location, frequency, instruments, quality assurance, procedural blanks, replicate samples.
- Registry of all relevant sample characteristics when available: brand, geographical and environmental origin, volumes, production dates, information on water treatment and additives.
- Particle extraction process specifications: sample volumes, chemicals used for digestion and density separation, type and pore size of filters.
- Spectral analysis:
  ○ Use of one of the currently validated methods: FTIR, RM, SEM, Pyr-GC-MS and SEM/EDS.
  ○ Proportion of extracted particles for analysis.
  ○ Spectral similarity index and which spectral libraries are used (bespoke or commercially available).
- Post-sampling handling: measures to protect cross-contamination and use of procedural blank samples in all experimental aspects to ensure effectiveness and account for experimental errors.
- Detailed reporting of all aspects of research including design, execution and statistical analysis.

In terms of future research there is a clear need for research on MP contamination of drinking water in countries beyond Europe where there is less data. Comparison between table water, natural mineral and spring waters to detect differences is another area that has not been explored. The additional exposure pathway via the use of MP contaminated water for incorporation into food also merits further research.

As this review shows, there are still relatively few studies examining MP contamination in drinking water, and levels vary significantly. The presence of MP in human stool samples has recently been verified [28], although the effects on human health are still under examination [28, 41, 111–113]. Given the amount of water humans drink and its use for incorporation into food, a clearer understanding of the levels of MP present in drinking water is needed, in order to better assess the risks that MPs in water present. Quantification of MPs human exposures is an integral part of the exposure assessment in the wider frame of a risk assessment to determine the likelihood of MPs having adverse human health effects [114, 115].

Our findings support the omnipresent MPs contamination of drinking water. Current food and drinking water safety regulation and standards around the world [49, 116, 117] adopt the precautionary principle [118, 119] on food safety risk management. The principle dictates that in the face of scientific uncertainty concerning possible harmful effects, after an initial assessment of available evidence has been completed and a comprehensive risk assessment is anticipated, risk management measures must be adopted in order to ensure the protection of health. The weight of the current evidence suggests that the time may have come to implement protective measures against the ingestion of MPs.

## Supporting information

S1 Checklist PRISMA checklist. (PDF)

S1 Protocol Systematic review protocol. Published in the International prospective register of systematic reviews (PROSPERO)
(PDF)

S1 Appendix Exclusion reasons during the second level screening. (PDF)

S1 Table Search strategy for MEDLINE (OVID). (PDF)

S2 Table Risk of bias (RoB) assessment tool template. (PDF)

S3 Table Study characteristics. (PDF)

S1 Fig Forest plot of TW studies random-effects model analysis. The x axis represents the standardized mean difference (SMD) expressed in MPs/L. The vertical line is the line of null effect where MP content is 0. The grey boxes represent the pooled effect estimate and the lines the CI 95%. The size of the boxes is proportional to the study weight. The diamond is the combined point estimate and CI for each of the subgroups.
(PDF)

S2 Fig Forest plot of BW studies random-effects model analysis. The x axis represents the standardized mean difference (SMD) expressed in MPs/L. The vertical line is the line of null effect where MP content is 0. The grey boxes represent the pooled effect estimate and the lines the CI 95%. The size of the boxes is proportional to the study weight.
(PDF)

S3 Fig Forest plot of BW studies random-effects model analysis excluding the high RoB study. The x axis represents the standardized mean difference (SMD) expressed in MPs/L. The vertical line is the line of null effect where MP content is 0. The grey boxes represent the pooled effect estimate and the lines the CI 95%. The size of the boxes is proportional to the study weight.
(PDF)

S4 Fig Forest plot of sub-group analysis of BW studies. Mixed-effects (plural model) analysis. The x axis represents the standardized mean difference (SMD) expressed in *MPs/*L. The vertical line is the line of null effect where MP content is 0. The grey boxes represent the pooled effect estimate and the lines the CI 95%. The size of the boxes is proportional to the study weight. The diamonds are the combined point estimates and CI for each of the subgroups. The red square is the overall pooled effect for all subgroups.
(PDF)
