# Supplementary material for: Microplastic contamination of drinking water: A systematic review
Source: PLoS One. 2020 Jul 31;15(7):e0236838. doi: 10.1371/journal.pone.0236838 (PMC7394398; doi:10.1371/journal.pone.0236838)
Supplement: S1 Protocol — Published in the International prospective register of systematic reviews (PROSPERO) (PDF) [file pone.0236838.s002.pdf]

Microplastics contamination of food intended for human consumption and drinking water: a systematic review

Evangelos Danopoulos, Lauren Jenner, Maureen Twiddy, Jeanette Rotchell

Citation

Evangelos Danopoulos, Lauren Jenner, Maureen Twiddy, Jeanette Rotchell. Microplastics contamination of food intended for human consumption and drinking water: a systematic review. PROSPERO 2019 CRD42019145290 Available from:
https://www.crd.york.ac.uk/prospero/display_record.php?ID=CRD42019145290

Review question

What is the occurrence of microplastics in food intended for human consumption, including drinking water, and what is the human exposure via the ingestion route?

Searches

The following online data bases/sources will be searched: MEDLINE (OVID interface, 1946 onwards), EMBASE (OVID interface, 1974 onwards). Medical Subject Headings (MeSH) and vocabulary thesaurus tools will be used for both sources. The Web of Science core collection (Wed of Science, 1900 onwards) will be searched as a multidisciplinary source.There will be no time frame for the studies, other than the date that the databases that we will use were launched. There will be no language and study design limits set for the searches.

In addition, in order to validate that all relevant studies have been included, the reference lists of the reviews that are discovered by the search will be searched, as well as the reference lists of relevant reports, that have already been published. Advice from experts in the field of microplastics will be sought to ensure that the results are inclusive.

The searches will be re-run before the final analysis to identify any further studies.

Types of study to be included

Only primary studies will be included. Commentaries, opinion pieces, proceedings of conferences, editorials and non peer reviewed reports will be excluded.

Condition or domain being studied

The human health effects may come from the microplastics causing physical or chemical damage. The effects can come from the plastics' primary components (polymers) or the additives that are added to polymers during the manufacturing process to enhance their attributes. Plastic has shown the ability to sorb persistent, bio accumulative and toxic substances which can later be leeched from it. Finally, microplastics have proven to be a good substrate to be colonized by microorganisms; effectively transporting them and dispersing them into novel environments. Nanoplastics have the ability to cross membranes possibly delivering substances to different locations than the aforementioned uptakes.

Participants/population

There are no restrictions placed on the population of study. The review scope is food and drinking water that can be consumed by any member of the population of the world.

Intervention(s), exposure(s)

The distribution of microplastics in the environment have been identified in varying concentrations and compositions in sea water fresh water, sediments, the atmosphere and in food. Whether their effects on human health are significant or not is yet to be established. The first step in doing that is establishing the exposure levels.

Microplastics can affect organisms via direct and indirect pathways. The documented effects to organisms, so far, reveal at least three different mechanisms of exposure, uptake and effect; ingestion, inhalation and

dermal absorption. The uptake route that will be investigated by the systematic review is ingestion.

Comparator(s)/control
Not applicable.

Context
The chemical composition of microplastics is synthetic polymers. Microplastics are defined as plastic particles less than 5 mm in size in any one dimension and Nanoplastics <100 nm.
Only studies that report on food samples as defined by Regulation (EC) No 178, 2002 "any substance or product, whether processed, partially processed or unprocessed, intended to be, or reasonably expected to be ingested by humans. 'Food' includes drink, chewing gum and any substance, including water, intentionally incorporated into the food during its manufacture, preparation or treatment" will be included.

Further inclusion criteria:

Studies reporting on samples that have not been collected as food but are regularly consumed as such.

Studies that look at the existence of microplastics in sea life regardless of the species of the organism or the part of the body that microplastics are reported to be found in.

Only commercially relevant species of sea life will be included (seafood).

If a study focuses on the GI tract of a type of seafood, it will only be included if the species of the seafood is small and it is reasonable to assume that it is usually eaten whole with the GI tract intact.

Studies must use one of the following four validated processes for the identification of microplastics: Fourier-transform infrared spectroscopy (FT-IR), Raman spectroscopy (RM), pyrolysis gas chromatography/ mass spectrometry (Pyr-GC-MS) and scanning electron microscopy plus energy-dispersive X-ray spectroscopy (SEM/EDS).

Studies must use procedural blank samples to validate that the samples have not been contaminated after their collection.

Main outcome(s)
Microplastics presence in the sample for the quantitative analysis and the microplastics content of the sample for the meta-analysis part of the review. For the quantitative analysis we will be looking at the mean value and the standard deviation in each type of sample in each paper. If the mean value is not provided but the individual data for the samples are available, we will calculate the values using the standard formulae for mean and standard variation. Different units of measurement might be used across the studies. Examples of different units include microplastics/ individual, microplastics/ g, microplastics/ ml. All different units will be extracted.

* Measures of effect

Not applicable.

Additional outcome(s)
Additional information of interest are the percentage of the sample (N) that was analysed for polymeric composition; the results of polymeric composition, expressed in percentage per N; and the method that was used for the extraction of the particles from the sample.

* Measures of effect

Not applicable.

Data extraction (selection and coding)
The screening of titles and abstracts of the identified papers will be done independently by two reviewers according the inclusion/ exclusion criteria. If there are any discrepancies between the reviewers, they will be resolved by a third-party arbitration. For the studies that meet the inclusion criteria the full papers will be downloaded for the second level screening.

The second level screening will be undertaken by the lead author, and he will record the reasons for excluding studies. 20% of the full text studies will also be screened by the second reviewer for validation of the process. The screening process will have two outcomes one for studies to be included in the narrative analysis and one for the meta-analysis. Additional criteria will be applied for the meta-analysis.

The data extraction process has been piloted by a scoping review. It will be carried out by one of the reviewers. The form that will be used has been developed, used and validated by the scoping review. The extraction procedure has also been validated during the scoping review. For the extraction of the data we will use an Excel file.

The data variables that will be extracted for the included studies are: geographic location of the sampling site/s, geographic coordinates of sampling site/s, date of sampling, sampling method, sample kind and type, number of samples, microplastics extraction procedure, visual identification method, composition identification method, percentage of sample undergone composition identification method, results of procedural blank samples, identified type of polymer, statistical test, microplastics' content of the sample. If the content of microplastics is not reported but only the presence of absence of microplastics this will also be extracted to use in the narrative review. If more than one type of sample is included in one paper the microplastics content will be extracted separately for each sample.

Risk of bias (quality) assessment

A tailor-made assessment tool (check list) was constructed according to the guidelines set by the Centre for Reviews and Dissemination. The quality of reporting section was developed according to the STROBE Statement—checklist regarding items that should be included in reports of observational studies and the recommendations of the Agency for Healthcare Research and Quality U.S. Department of Health and Human Services.

In addition we also took into consideration the principles laid by the Environmental-Risk of Bias Too which has been adapted from the Cochrane Collaboration's tool for assessing risk of bias in randomised trials.

The rating of the studies will be: high risk, low risk or unclear risk of bias. The results of the quality assessment will be used to inform the findings of the systematic review regarding both the qualitative and the quantitative synthesis. The characteristics of the studies that will be evaluated by the checklist are:

Appropriateness of study design to the research objective

Sample method, location and randomisation.

Use of procedural blank samples

Use of replicate samples

Particles extraction method

Particles identification method

Amount of sample analysed for composition.

Particle composition match to the library of choice

Library of choice (type, kind)

Statistical analysis

Interpretation

Quality of reporting

Methodology

Limitations

Sampling size

Strategy for data synthesis

If the studies are homogenous enough, we will undertake a meta-analysis. The homogeneity of the studies will be judged in terms of the overall design of the study, focusing on sample type, the unit of measurement used to present the results and the statistical analysis used. The results of the meta-analysis will be presented as weighed mean with standard deviations (SD). If the data are not presented in the studies using mean and SD, we will use the standard formulae for computing these values, if the date to do so are available. If there are missing data we will contact the authors. If the authors do not provide the data, the study will not be included in the meta-analysis, and it will be clearly stated in the report. The assessment of heterogeneity will be done by comparing the outcomes of the studies visually using Forest plots, and tested statistically using the Higgins I^2 test. If the heterogeneity is considerable (Higgins I^2 over 75%), we may undertake a qualitative analysis instead of a meta-analysis. All included studies will be reported in a narrative analysis. For the meta-analysis we will use the formulae for combining groups proposed by the Cochrane handbook. For the computation of the results we will use the software excel (Microsoft) and R (version 3.5.0, 2018-04-23), where it is appropriate.

For the qualitative analysis we will use a narrative synthesis approach according to the guidelines set by the Centre for Reviews and Dissemination. The primary and the additional outcomes of the studies, as described above, will be summarized and presented in tables and comparisons will be made between them. The studies will be grouped according to their samples. The risk of bias assessment will be taken into consideration for the weight of the evidence in the narrative synthesis procedure.

Analysis of subgroups or subsets

Not applicable.

Contact details for further information

Evangelos Danopoulos
hyen7@hyms.ac.uk

Organisational affiliation of the review

Hull York Medical School, University of Hull, University of York
https://www.hyms.ac.uk

Review team members and their organisational affiliations

Mr Evangelos Danopoulos. Hull York Medical School, University of Hull, University of York
Miss Lauren Jenner. Hull York Medical School, University of Hull, University of York
Dr Maureen Twiddy. Hull York Medical School, University of Hull, University of York
Professor Jeanette Rotchell. University of Hull

Type and method of review

Meta-analysis, Narrative synthesis, Systematic review

Anticipated or actual start date

10 July 2019

Anticipated completion date

20 December 2019

Funding sources/sponsors

The project is funded through a University of Hull PhD Scholarship.

Conflicts of interest

Language
English

Country
England

Stage of review
Review Ongoing

Subject index terms status
Subject indexing assigned by CRD

Subject index terms
Drinking Water; Food; Humans; Plastics; Water Supply

Date of registration in PROSPERO
27 August 2019

Date of publication of this version
27 August 2019

Details of any existing review of the same topic by the same authors

Stage of review at time of this submission

Stage	Started	Completed
Preliminary searches	Yes	No
Piloting of the study selection process	Yes	Yes
Formal screening of search results against eligibility criteria	No	No
Data extraction	No	No
Risk of bias (quality) assessment	No	No
Data analysis	No	No

The record owner confirms that the information they have supplied for this submission is accurate and complete and they understand that deliberate provision of inaccurate information or omission of data may be construed as scientific misconduct.

The record owner confirms that they will update the status of the review when it is completed and will add publication details in due course.

Versions
27 August 2019

PROSPERO
This information has been provided by the named contact for this review. CRD has accepted this information in good faith and registered the review in PROSPERO. The registrant confirms that the information supplied for this submission is accurate and complete. CRD bears no responsibility or liability for the content of this registration record, any associated files or external websites.
