# Supplementary material for: Microplastic contamination of drinking water: A systematic review
Source: PLoS One. 2020 Jul 31;15(7):e0236838. doi: 10.1371/journal.pone.0236838 (PMC7394398; doi:10.1371/journal.pone.0236838)
Supplement: S1 Appendix — (PDF) [file pone.0236838.s003.pdf]

S1 Appendix. Exclusion reasons during the second level screening.

- For all food themes:

30: focused only on the GI tract of the seafood, for seafood that is not eaten whole

26: MPs identification method was not one of the four accepted in this SR

15: did not use procedural blanks samples

12: the sample was not food or drinking water

9: studies were not available

8: studies were not environmental study

4: the studies did not mention any MPs content data

4: the results were not specific to MPs

3: papers reporting conferences

1: was a duplicate publication

1: was a corrigendum to a study that is already included in the review

- For drinking water only:

3: the sample was not drinking water

2: studies were not available

2: studies were not environmental study

1: MPs identification method was not one of the four accepted in this SR

- For rerun of searches for drinking water only

2: not drinking water

1: study was not environmental study

1: MPs identification method was not one of the four accepted in this SR

1: not primary research
