# Supplementary material for: Microplastic contamination of drinking water: A systematic review
Source: PLoS One. 2020 Jul 31;15(7):e0236838. doi: 10.1371/journal.pone.0236838 (PMC7394398; doi:10.1371/journal.pone.0236838)
Supplement: S1 Table — (PDF) [file pone.0236838.s004.pdf]

S1 Table. Search strategy for MEDLINE (OVID) with MeSH for all available food categories.

1	microplastic*
2	micro-plastic*
3	nanoplastic*
4	nano-plastic*
5	plastic/
6	micro
7	fiber*
8	fibre
9	microfiber*
10	microfibre*
11	micro-fiber*
12	micro-fibre*
13	particle*
14	particle size/
15	pellet*
16	fragment*
17	film*
18	filament*
19	rubber/
20	5 and 6
21	5 and 7
22	5 and 8
23	5 and 9
24	5 and 10
25	5 and 11
26	5 and 12
27	5 and 13
28	5 and 14
29	5 and 15
30	5 and 16
31	5 and 17
32	5 and 18
33	5 and 19
34	1 or 2 or 3 or 4 or 20 or 21 or 22 or 23 or 24 or 25 or 26 or 27 or 28 or 29 or 30 or 31 or 32 or 33
35	food quality/ or food dye/ or food ingredient/ or canned food/ or food packaging/ or food contamination/ or food industry/ or food insecurity/ or cooked food/ or food safety/ or food analysis/ or food chain/ or fast food/ or dried food/ or sea food/ or food handling/ or food security/ or food/
36	water table/ or drinking water/ or water quality/ or tap water/ or water pollutant/ or water contamination/ or water pollution/
37	sea food/
38	fish/
39	bivalve disease/ or bivalve/
40	Crustacea/
41	35 or 36 or 37 or 38 or 39 or 40

42	34 and 41
43	nanoparticle/ or nano
44	5 and 43
45	41 and 44
46	42 or 45
