# Supplementary material for: Microplastic contamination of drinking water: A systematic review
Source: PLoS One. 2020 Jul 31;15(7):e0236838. doi: 10.1371/journal.pone.0236838 (PMC7394398; doi:10.1371/journal.pone.0236838)
Supplement: S2 Table — (PDF) [file pone.0236838.s005.pdf]

S2 Table. Risk of bias (RoB) assessment tool template

**Domain**	**No**	**Question**	**Answer**	**Notes**	**Rating (high, low, unclear)**
**Internal validity**					
Appropriateness of study design to the research objective	1	Is the design appropriate for the questions of the study?			
**Sampling**					
Sample method	2	Has the method been used in other studies?			
	3	Is the method validated?			
	4	Are there precautions in place to protect further contamination of the sample?			
Sample location	5	Is there a rationale available?			
	6	Is the location appropriate?			
Sample randomization	7	Is the sampling method guarantying randomization of the sample?			
Use of procedural blank samples	8	Are the results of the procedural blank samples reported?			
Use of replicate samples	9	Is the study using replicate samples?			
	10	How many?			
**Analysis**					
Particles extraction method	11	Is the method used by other studies?			
	12	Is the method validated?			
Particles identification method	13	Is the method one of the four validated methods?			
Amount of sample analysed for composition.	14	How much of the sample has been analysed?			
Particle composition match to the library of choice	15	Is the match > or < 60% match?			
Library of choice (type, kind)	16	Is the library made by the lab or is it a commercial library?			

	17	Is one library or more being used?			
Statistical analysis	18	Is the statistical analysis appropriate for the sample?			
Interpretation	19	Has the interpretation of the results been based on the outcomes of the analysis?			
**Quality of reporting**					
Methodology	20	Have the methods used in the study been reported in detail?			
Limitations	21	Have the study recognized limitations?			
				**overall score**	
