# Supplementary material for: Microplastic contamination of drinking water: A systematic review
Source: PLoS One. 2020 Jul 31;15(7):e0236838. doi: 10.1371/journal.pone.0236838 (PMC7394398; doi:10.1371/journal.pone.0236838)
Supplement: S3 Table — (PDF) [file pone.0236838.s006.pdf]

S3 Table. Study characteristics.

Study	Year	Geographic location	Sample	N	MPs extraction procedure	MPs identification method	Reported outcome
Mason et al. [1]	2018	Brazil, China France, Germany, India, Indonesia, Italy, Lebanon, Mexico, UK, USA.	BW: table and natural mineral	N=259 bottles<br>11 brands: 9 brands 500–600 ml per bottle, 2 brands 0.75–2 L per bottle<br>n=253 plastic bottles<br>n=6 glass bottles	Their own procedure	FTIR<br>(particles > 100 µm)	Mean MPs content per volume
Mintenig et al. [2]	2019	Germany	TW: Groundwater from wells	N=24 samples<br>n=9 raw (8 m^3)<br>n=15 drinking (32 m^3)	Mintenig et al. [3]	m-FTIR	Mean MPs content per volume and frequency of occurrence
Kankanige and Babel [4]	2020	Thailand	BW: Spring and tap	N=95<br>n=65 PET single use (still)<br>n=30 glass (carbonated)<br>(10 brands, total 43.23 L)	Maes et al. [5]	FTIR and RM	Mean MPs content per volume with SD
Oßmann et al. [6]	2018	Germany	BW: mineral	N=32<br>n=12 PET reusable n=10 PET single use n=9 glass reusable<br>n=1 glass single use<br>(21 brands, 0.5 – 1 L per bottle)	Oßmann et al. [7]	m-RM	Mean MPs content per volume with SD
Pivokonsky et al.[8]	2018	Czech Republic	TW: WTPs[a] from open reservoirs	N=36<br>(1 L per sample)	Anderson et al. [9], Leslie et al. [10] and Mintenig et al. [3]	m-FTIR for particles >10 µm<br>m-RM for particles 1–10 µm	Mean MPs content per volume with SD

Schymanski et al. [11]	2018	Germany	BW: mineral	N=38<br>n=15 returnable plastic bottles<br>n=11 single-use plastic bottles<br>n=3 beverage cartons<br>n=9 glass bottles<br>(volume range 700-1500 ml)	their own method	m-RM	Mean MPs content per volume with SD
Shruti et al. [12]	2020	Mexico	TW:	N=42<br>(3 L x 3 *per site*)	Liebezeit and Liebezeit [13], Kosuth et al. [14], Schymanski et al. [11]	m-RM	Mean MPs content per volume with SD
Strand et al. [15]	2018	Denmark	TW	N=17<br>n=9 private households<br>n=3 private workplace<br>n=5 private or public institutions	Strand et al. [16]	m-FTIR	Frequency of occurrence
Tong et al. [17]	2020	China	TW	N=38<br>(2 L *per site*)	their own procedure	RM	Mean MPs content per volume with SD
Wiesheu et al. [18]	2016	Germany	BW: mineral	n=1 water<br>(3 L)	their own procedure	m-RM	MPs content range per volume
Zhang et al. [19]	2020	China	TW	N=7<br>(4.5 L x 3 per site)	their own procedure	m-FTIR	Mean MPs content per volume with SD
Zuccarello et al. [20]	2019	Italy	BW: Mineral still and sparkling	N=10<br>(10 brands, 500 ml per bottle)	their own procedure	SEM-EDX	Mean MPs content per volume with SD

[a] Water treatment plants

References

1. Mason SA, Welch VG, Neratko J. Synthetic polymer contamination in bottled water. Frontiers in Chemistry. 2018;6. doi: 10.3389/fchem.2018.00407. PubMed PMID: 30255015.
2. Mintenig SM, Loder MGJ, Primpke S, Gerdts G. Low numbers of microplastics detected in drinking water from ground water sources. The Science of the total environment. 2019;648:631-5. doi: 10.1016/j.scitotenv.2018.08.178. PubMed PMID: 30121540.
3. Mintenig SM, Int-Veen I, Loeder MGJ, Primpke S, Gerdts G. Identification of microplastic in effluents of waste water treatment plants using focal plane array-based micro-Fourier-transform infrared imaging. Water Research. 2017;108:365-72. doi: 10.1016/j.watres.2016.11.015. PubMed PMID: 27838027.
4. Kankanige D, Babel S. Smaller-sized micro-plastics (MPs) contamination in single-use PET-bottled water in Thailand. Science of the Total Environment. 2020;717:137232. doi: 10.1016/j.scitotenv.2020.137232. PubMed PMID: 32062244.
5. Maes T, Jessop R, Wellner N, Haupt K, Mayes AG. A rapid-screening approach to detect and quantify microplastics based on fluorescent tagging with Nile Red. Scientific Reports. 2017;7(1):44501. doi: 10.1038/srep44501. PubMed PMID: 28300146.
6. Oßmann BE, Sarau G, Holtmannspötter H, Pischetsrieder M, Christiansen SH, Dicke W. Small-sized microplastics and pigmented particles in bottled mineral water. Water Research. 2018;141:307-16. doi: 10.1016/j.watres.2018.05.027. PubMed PMID: 29803096.
7. Oßmann BE, Sarau G, Schmitt SW, Holtmannspoetter H, Christiansen SH, Dicke W. Development of an optimal filter substrate for the identification of small microplastic particles in food by micro-Raman spectroscopy. Analytical and Bioanalytical Chemistry. 2017;409(16):4099-109. doi: 10.1007/s00216-017-0358-y. PubMed PMID: 28439620.
8. Pivokonsky M, Cermakova L, Novotna K, Peer P, Cajthaml T, Janda V. Occurrence of microplastics in raw and treated drinking water. The Science of the Total Environment. 2018;643:1644-51. doi: 10.1016/j.scitotenv.2018.08.102. PubMed PMID: 30104017.
9. Anderson PJ, Warrack S, Langen V, Challis JK, Hanson ML, Rennie MD. Microplastic contamination in Lake Winnipeg, Canada. Environmental Pollution. 2017;225:223-31. doi: 10.1016/j.envpol.2017.02.072. PubMed PMID: 28376390.
10. Leslie HA, Brandsma SH, van Velzen MJM, Vethaak AD. Microplastics en route: Field measurements in the Dutch river delta and Amsterdam canals, wastewater treatment plants, North Sea sediments and biota. Environment international. 2017;101:133-42. doi: 10.1016/j.envint.2017.01.018. PubMed PMID: 28143645.
11. Schymanski D, Goldbeck C, Humpf HU, Furst P. Analysis of microplastics in water by micro-Raman spectroscopy: Release of plastic particles from different packaging into mineral water. Water Research. 2018;129:154-62. doi: 10.1016/j.watres.2017.11.011. PubMed PMID: 29145085.
12. Shruti VC, Perez-Guevara F, Kutralam-Muniasamy G. Metro station free drinking water fountain- A potential "microplastics hotspot" for human consumption. Environmental Pollution. 2020;261. doi: 10.1016/j.envpol.2020.114227.
13. Liebezeit G, Liebezeit E. Synthetic particles as contaminants in German beers. Food Additives & Contaminants: Part A. 2014;31(9):1574-8. doi: 10.1080/19440049.2014.945099.
14. Kosuth M, Mason SA, Wattenberg EV. Anthropogenic contamination of tap water, beer, and sea salt. PLOS ONE. 2018;13(4):e0194970. doi: 10.1371/journal.pone.0194970.
15. Strand J, Feld L, Murphy F, Mackevica A, Hartmann NB. Analysis of microplastic particles in Danish drinking water. DCE-Danish Centre for Environment and Energy, 2018 877156358X.
16. Strand J, Dahl K, Boutrup SJNfD-NCfMoEAU, Institut for Bioscience. Forslag til målemetode til brug for undersøgelser af mikroplast i taphanevand. 2018.
17. Tong HY, Jiang QY, Hu XS, Zhong XC. Occurrence and identification of microplastics in tap water from China. Chemosphere. 2020;252. doi: 10.1016/j.chemosphere.2020.126493.

18. Wiesheu AC, Anger PM, Baumann T, Niessner R, Ivleva NP. Raman microspectroscopic analysis of fibers in beverages. Analytical Methods. 2016;8(28):5722-5. doi: 10.1039/c6ay01184e.
19. Zhang M, Li JX, Ding HB, Ding JF, Jiang FH, Ding NX, et al. Distribution Characteristics and Influencing Factors of Microplastics in Urban Tap Water and Water Sources in Qingdao, China. Analytical Letters. 2020;53(8):1312-27. doi: 10.1080/00032719.2019.1705476.
20. Zuccarello P, Ferrante M, Cristaldi A, Copat C, Grasso A, Sangregorio D, et al. Exposure to microplastics (<10mum) associated to plastic bottles mineral water consumption: The first quantitative study. Water Research. 2019;157:365-71. doi: 10.1016/j.watres.2019.03.091. PubMed PMID: 30974285.
