# Supplementary figures and images for: Microplastic contamination of drinking water: A systematic review
Source: PLoS One. 2020 Jul 31;15(7):e0236838. doi: 10.1371/journal.pone.0236838 (PMC7394398; doi:10.1371/journal.pone.0236838)

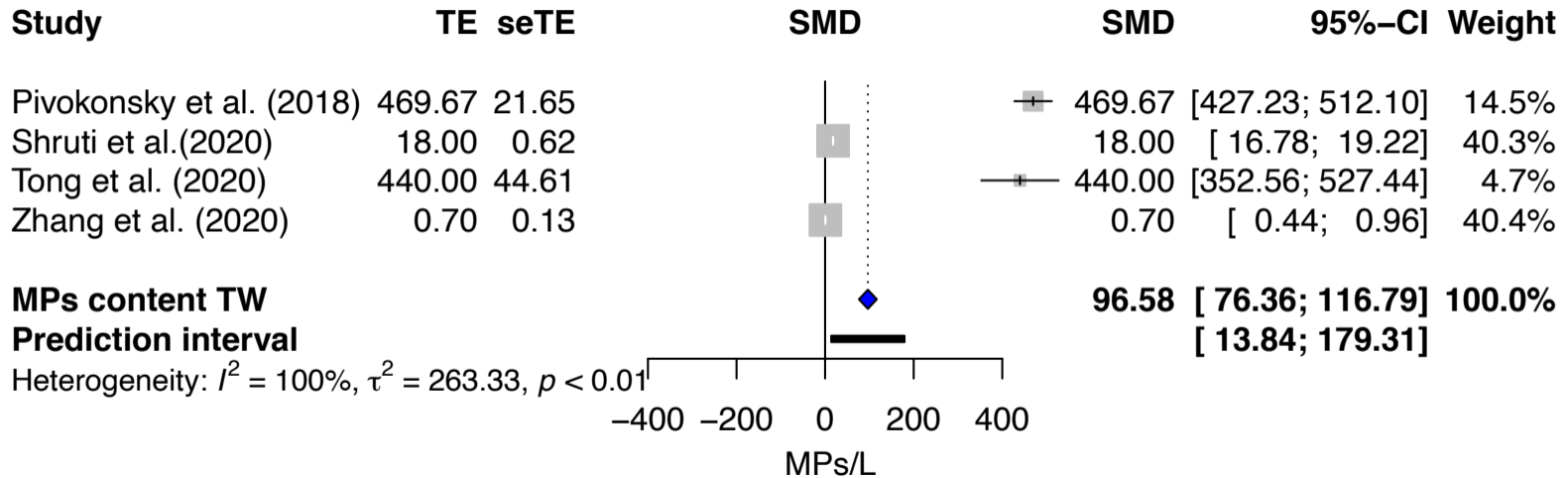

Supplement: S1 Fig — The x axis represents the standardized mean difference (SMD) expressed in MPs/L. The vertical line is the line of null effect where MP content is 0. The grey boxes represent the pooled effect estimate and the lines the CI 95%. The size of the boxes is proportional to the study weight. The diamond is the combined point estimate and CI for each of the subgroups. (PDF) [file pone.0236838.s007.pdf]

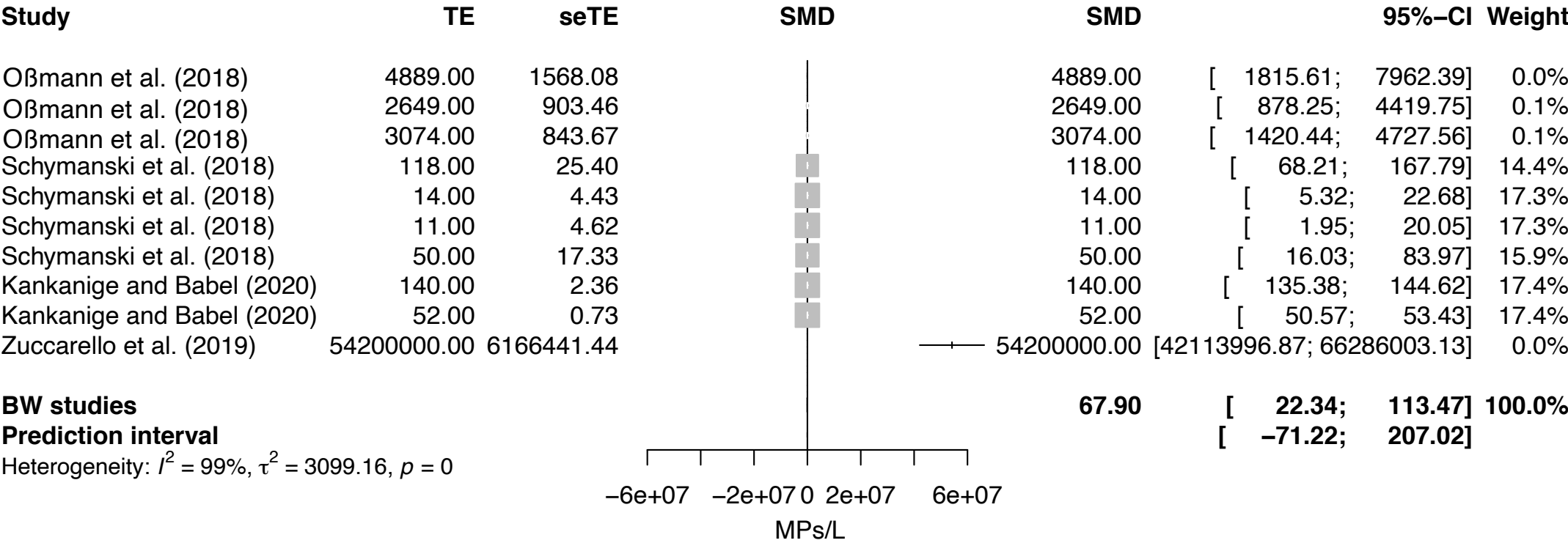

Supplement: S2 Fig — The x axis represents the standardized mean difference (SMD) expressed in MPs/L. The vertical line is the line of null effect where MP content is 0. The grey boxes represent the pooled effect estimate and the lines the CI 95%. The size of the boxes is proportional to the study weight. (PDF) [file pone.0236838.s008.pdf]

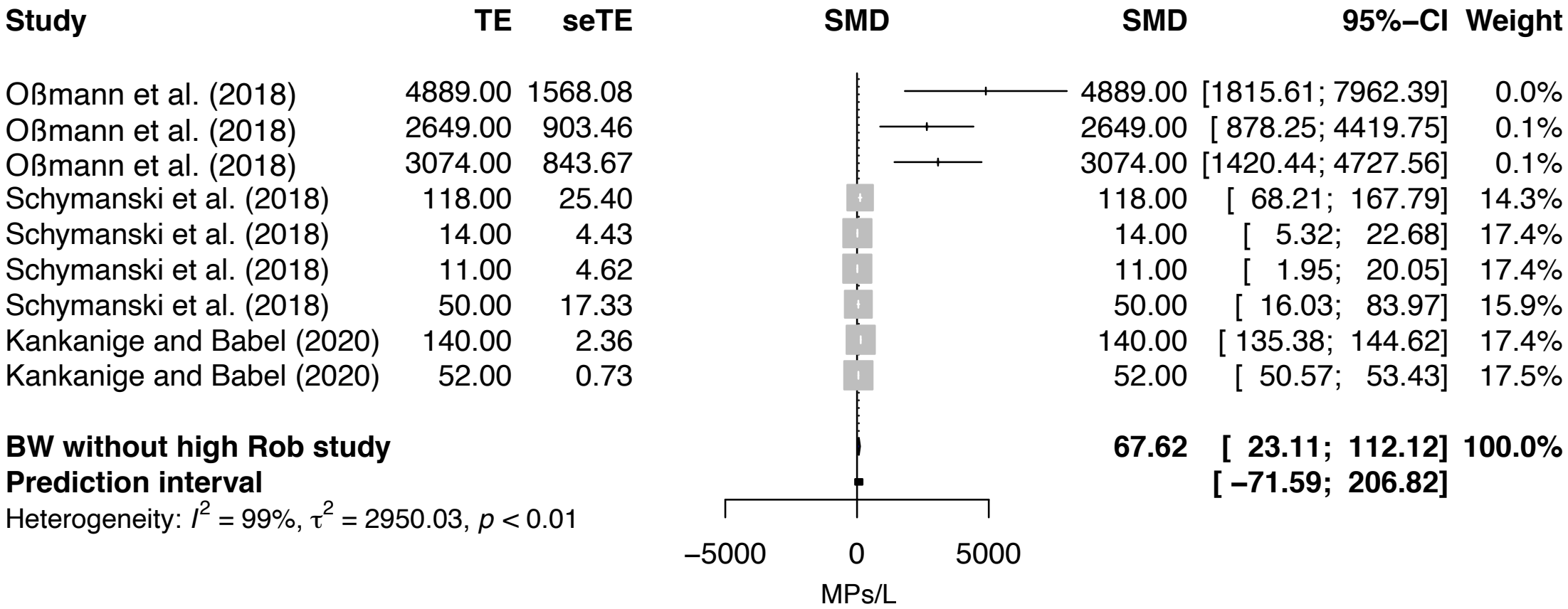

Supplement: S3 Fig — The x axis represents the standardized mean difference (SMD) expressed in MPs/L. The vertical line is the line of null effect where MP content is 0. The grey boxes represent the pooled effect estimate and the lines the CI 95%. The size of the boxes is proportional to the study weight. (PDF) [file pone.0236838.s009.pdf]

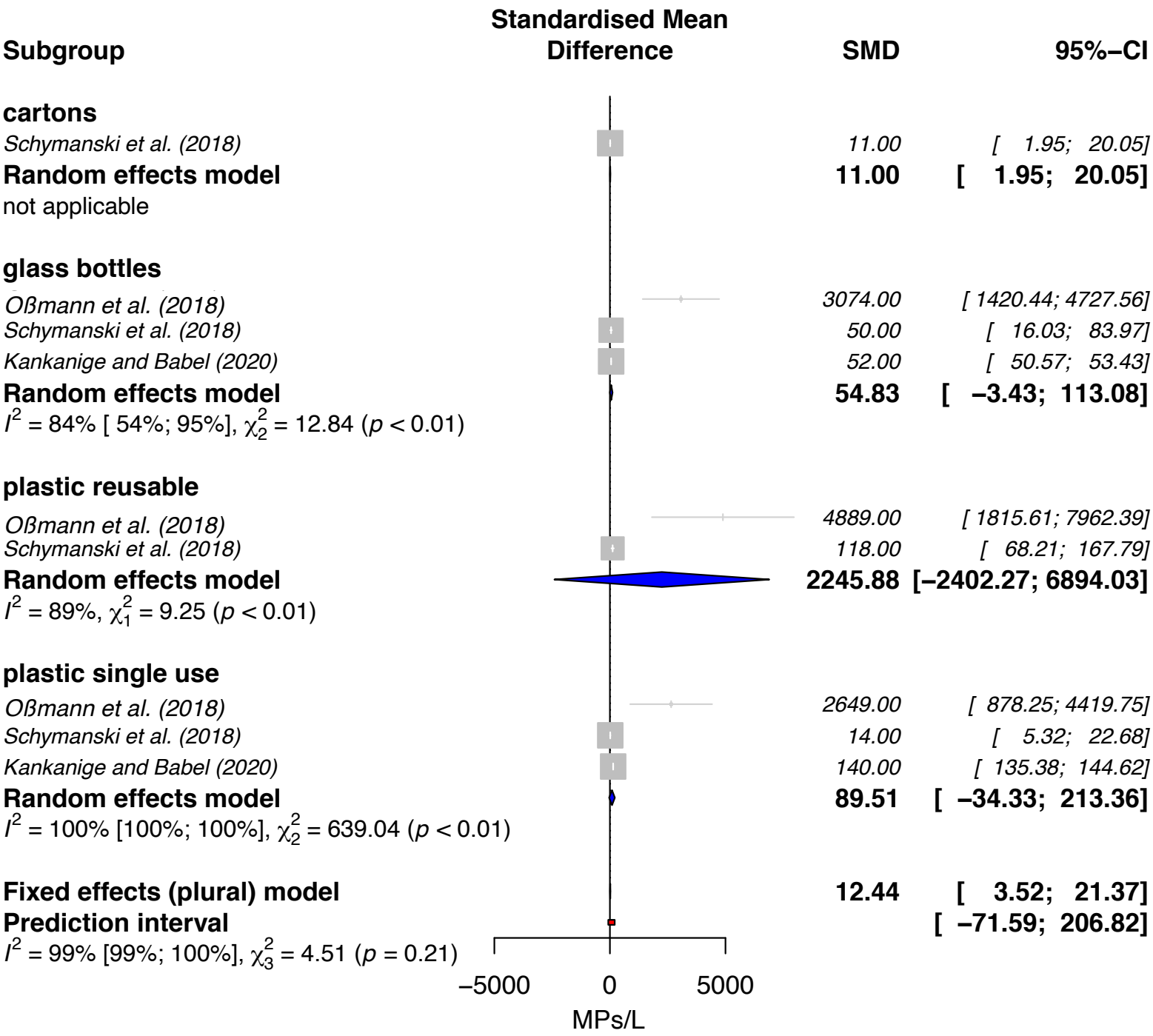

Supplement: S4 Fig — Mixed-effects (plural model) analysis. The x axis represents the standardized mean difference (SMD) expressed in MPs/L. The vertical line is the line of null effect where MP content is 0. The grey boxes represent the pooled effect estimate and the lines the CI 95%. The size of the boxes is proportional to the study weight. The diamonds are the combined point estimates and CI for each of the subgroups. The red square is the overall pooled effect for all subgroups. (PDF) [file pone.0236838.s010.pdf]
